# Work-Life Conflict, Burnout, and Associated Factors Among Hydroelectric Power Plant Employees: A Cross-Sectional Study in Turkey

**DOI:** 10.7759/cureus.64425

**Published:** 2024-07-12

**Authors:** İrem Medeni, Volkan Medeni, Osman Burak Demirbaş, Mustafa Necmi İlhan

**Affiliations:** 1 Employee Health Department, General Public Health Directorate, Ministry of Health, Ankara, TUR; 2 Department of Occupational Medicine, Faculty of Medicine, Gazi University, Ankara, TUR; 3 Department of Public Health, Faculty of Medicine, Gazi University, Ankara, TUR

**Keywords:** burnout, workplace conditions, psychosocial factors, work-life balance, power plant, occupational health and safety

## Abstract

Introduction: Power plants are associated with numerous occupational health and safety risk factors, with psychosocial risks being particularly significant. This study examines work-life conflict and burnout among power plant employees and discusses the factors associated with these issues.

Materials and methods: This cross-sectional study focused on employees at three hydroelectric power plants in Turkey. The inclusion criteria included employees with at least one year of tenure. Using cluster sampling, three plants were selected in Adana, Ankara, and Samsun. The sample size was determined to be 262, and 201 employees participated, yielding a 76.7% response rate. Data were collected via face-to-face interviews using a structured questionnaire, which encompasses the sub-dimensions of a valid and reliable scale: The Work-Life Conflict and Burnout sub-dimensions of the Copenhagen Psychosocial Questionnaire-III (COPSOQ-III) were used to measure the dependent variables. The independent variables included age, education level, total and weekly working hours, perceived health status, and department. The dependent variables were work-life conflict and burnout. Ethical approval was obtained from the Gazi University Ethics Committee. Statistical analysis compared the Pearson chi-square test, Fisher's exact test, and Yates correction with a significance threshold of p < 0.05.

Results: The mean age was 40.83 years, with an average tenure of 11.54 years and a weekly work time of 43.51 hours. Most participants (94.5%) were male; technical unit workers comprised 71.6%. Health issues included smoking (39.8%) and chronic diseases (19.9%). Concerns about the working environment include insufficient knowledge about safety (25.4%) and lack of knowledge about risk assessments (32.3%). Many workers reported lacking personal protective equipment (11.4%) and rest areas (15.4%). Negative health impacts from work were noted by 31.8%. In addition, 51.2% believed that noise levels were outside the acceptable range. Two-thirds of employees reported inadequate measures against physical risks in the workplace. Many participants experienced work-life conflict (13.9%) and burnout (14.5%). High work-life conflict was significantly associated with younger age groups, less tenure, and negative perceived health status. Burnout was significantly related to the duration of employment, weekly working hours, and perceived health status.

Conclusion: The study highlights the seriousness of burnout and work-life conflict among hydropower plant workers, emphasizing the need for administrative and organizational interventions to alleviate these issues. Regular occupational health and safety training, involvement in risk assessments, fair workload distribution, supportive work environments, and counseling services are recommended to reduce burnout and improve work-life balance.

## Introduction

Energy is an essential resource for human survival and the development of societies [1]. Due to the rapid depletion of traditional energy sources, hydroelectric power plants are becoming increasingly prevalent worldwide. Like all power plants, the primary purpose of hydroelectric power plants is to produce uninterrupted, reliable, efficient, economical, and environmentally friendly sustainable electrical energy [2].

Globally, most electrical energy is produced in hydroelectric power plants, including in Turkey. Sixty-five countries generate 50% of their national electricity production from hydroelectric power, 32 countries generate 80%, and 13 countries rely almost entirely on water power [3]. The hydroelectric industry employs 2.2 million people, with predictions that this number will rise to 3.7 million by 2050. More than half of the employment in this sector is concentrated in China and India, with Turkey ranking among the top 10 countries in terms of workforce in the hydroelectric industry [4].

Hydroelectric power plants are among the most hazardous workplaces. Numerous risks and hazards arise during operations [5]. Ensuring workplace health and safety is crucial for employees, as neglect can lead to accidents and fatalities [6]. While accidents in hydroelectric power plants are rare, their consequences can be severe [7]. The primary occupational health and safety hazards in these plants include moving machinery, excessive physical exertion, working at heights, dust exposure, indoor work, and risks of slipping and falling [8].

Additional hazards involve the unexpected release of flammable and explosive energy, air pollution from toxic chemicals and gases, electrical cables, switchgear, cooling systems, and the extensive use of hydraulic oils in transformers. Noise pollution is another significant concern [9]. Furthermore, exposure to extremely low-frequency electromagnetic fields is inherent in hydroelectric power plants and poses potential risks, such as stress, depression, anxiety, poor sleep quality, and eventually burnout. These adverse effects are likely due to prolonged exposure to magnetic fields, elevated oxidative stress markers, and challenging working conditions, all of which contribute to the development of burnout syndrome [10,11].

However, a review of the literature reveals a scarcity of studies examining work-life conflict and burnout among power plant employees, and those available have involved only a small number of participants.

By focusing on the interplay between personal characteristics, working conditions, work-life conflict, and burnout, our research seeks to provide a comprehensive understanding of the occupational health challenges faced by employees in this sector. The findings are expected to contribute to the development of strategies for mitigating psychosocial risks and improving overall employee health and safety in hydroelectric power plants.

Occupational health and safety risks in hydroelectric power plants significantly impact the well-being of employees, with psychosocial risks playing a crucial role. This study aims to examine the personal characteristics of employees in hydroelectric power plants, investigate their working conditions, evaluate their experiences of work-life conflict and burnout, and discuss the relationships between these factors and their determinants.

## Materials and methods

This research is a cross-sectional study conducted in three hydroelectric power plants affiliated with the Electricity Generation Corporation, located in Adana, Ankara, and Samsun, Turkey. The Electricity Generation Corporation is a public institution established to generate electricity and is responsible for the majority of the country's electricity production. This company operates 44 hydroelectric power plants.

The selection of Adana, Ankara, and Samsun for this research was based on several strategic considerations. These cities were chosen due to their geographical diversity, which comprehensively represents different regions in Türkiye. Adana, located in the southern part of the country, represents the Mediterranean region. Ankara, the capital city, is situated in the central Anatolian region, while Samsun, located on the northern coast, represents the Black Sea region. This geographical spread ensures that the study encompasses a wide variety of environmental, operational, and socioeconomic conditions, which is crucial for obtaining a well-rounded understanding of the working conditions and challenges employees face in different hydroelectric power plants across Türkiye. In addition, these cities were selected because they host significant hydroelectric power plants with many employees, ensuring that the study could achieve the required sample size and statistical power.

The sample size calculation was based on a power analysis: The effect size is a measure of the strength of the relationship between two variables. For this study, an effect size (w) of 0.2 was chosen, which corresponds to a small effect size as per Cohen's conventions. The significance level, set at 0.05, represents the probability of rejecting the null hypothesis when it is actually true (type I error). The power of the test, set at 0.90, indicates a 90% chance of correctly rejecting the null hypothesis when it is false (1 - β, where β is the probability of a type II error). For a chi-square test with one degree of freedom, the degrees of freedom are calculated as the number of categories minus one. In this study, df = 1.

Using these parameters, the sample size was calculated using the following formula for a chi-square test: n = [(Z_1−α/2_+Z_1−β_ )^2^×(1−P_0_) / P_0_] x 1/w^2^

Where:

Z_1−α/2 _is the critical value of the standard normal distribution at α/2 (for a two-tailed test).

Z_1−β_ is the critical value of the standard normal distribution at β.

P_0_ is the assumed proportion under the null hypothesis.

w is the effect size.

Given:

Z_1−α/2_ = 1.96 (for α = 0.05)

Z_1−β_ = 1.28 (for β = 0.10)

w = 0.2

Plugging in these values, we calculate:

n = [(1.96+1.28)^2^×(1−0.5) / 0.5] x 1/0.2^2^

n = [(3.24)^2^×(0.5) / 0.5] x 1/0.04

n = [10.4976] × 25

n = 262.44

The minimum required sample size was 262. Utilizing a cluster sampling approach, we selected three hydroelectric power plants in three cities, each plant representing a cluster: Çatalan Hydroelectric Power Plant in Adana, Sarıyar Hydroelectric Power Plant in Ankara, and Suat Uğurlu Hydroelectric Power Plant in Samsun. These power plants were chosen from those with over 50 employees, aiming to encompass the entire population without specific sample selection. The inclusion criteria included employees with at least one year of tenure in these power plants. The workforce distribution across these plants was as follows: Çatalan (48), Sarıyar (68), and Suat Uğurlu (85), totaling 201 employees meeting the study's tenure criteria. The response rate was 76.7%.

We developed a data collection form based on a comprehensive literature review, consisting of 28 questions divided into two sections. It included a total of 28 questions. The first section focused on gathering descriptive information, while the second section delved into aspects of occupational health and safety. The data form encompassed inquiries regarding gender, age, educational attainment, working hours, departmental affiliation, smoking habits, presence of chronic ailments, utilization of sick leave, perceived health status, work-life balance, burnout status, physical hazards in the workplace environment, participation in occupational health and safety training, and the availability of rest areas at work.

Employees were also queried about various concerns, including their awareness of the outcomes of workplace environment assessments, participation in risk assessment studies, and the regular maintenance of work equipment. Furthermore, the questionnaire addressed employees' perceptions of the adequacy of measures taken against physical risk factors and their experiences with acquiring personal protective equipment.

The study investigated several independent variables, such as age groups, graduation status, total working time, weekly working time, perceived health status, and working department. These variables were assessed in their association with the dependent variables of work-life conflict and burnout status experienced by the participants

The study examined work-life conflict and burnout using questions from the Work-Life Conflict and Burnout sub-dimensions of the Copenhagen Psychosocial Questionnaire-III (COPSOQ-III) [12], a research tool for assessing workplace psychosocial conditions. The COPSOQ-III comprises 25 dimensions and 87 items. All dimensions, except job satisfaction, are scored on a five-point Likert scale. The work-life conflict dimension comprises five questions, and the burnout dimension comprises four questions, both scored on a five-point Likert scale: always (100), often (75), sometimes (50), rarely (25), and never/almost never (0). Higher scores indicate greater burnout. In our study, these two variables were categorized into four quartiles. In addition, the median value served as the cutoff point, classifying scores as high or low. The scale was translated into Turkish by Şahan et al., and this version has been deemed reliable and valid [13].

In assessing participants' perceived health status, they were asked, "How would you rate your overall health?" Responses were evaluated using a five-point Likert scale. Categories such as "moderate," "poor," and "very poor" were considered indicative of negative health perception.

The inquiry "Does the employer implement adequate precautions against physical risks?" was posed with a binary response format.

This study received approval from the Gazi University Ethics Committee on July 19, 2022 (2022-887). Following the acquisition of institutional permissions, data collection occurred in August 2022. Trained occupational safety experts from power plants conducted face-to-face interviews to complete the forms. The survey aimed to gauge perceptions regarding employer safety measures.

We inputted the gathered data from our study into the IBM SPSS Statistics for Windows, version 26.0 (released 20129, IBM Corp., Armonk, NY) and conducted statistical analyses using this software. Descriptive statistics were employed to present categorical variables in terms of numbers and percentages, while numerical variables were characterized using the mean, standard deviation, and median (with minimum-maximum values). To compare categorical variables, we utilized the Pearson chi-square test. In instances of small sample sizes where Pearson's chi-square test might produce unreliable outcomes, we applied Fisher's exact test along with Yates correction. Our chosen threshold for statistical significance was set at p < 0.05.

## Results

A total of 201 individuals, each with a minimum of one year of experience working in hydroelectric power plants, participated in this study. The mean age of the participants was 40.83 years with a standard deviation of 8.82 (range: 21 to 63). On average, the participants had been employed at their current workplace for 11.54 years with a standard deviation of 8.78 (range: 1 to 40). The average weekly working time was 43.51 hours with a standard deviation of 2.39 (range: 40 to 49).

Table 1 illustrates the distribution of descriptive characteristics among the participants. The majority of participants, comprising 94.5%, were male, and 74.1% fell within the age range of 30-49. Only 15.4% possessed a university degree or higher qualification. Among the respondents, 71.6% worked in technical units. Approximately 29.9% of the participants had been employed at their current workplace for less than five years. Those who worked more than 40 hours per week constituted 69.2% of the sample. Smokers represented 39.8% of the participants, while 19.9% reported having chronic diseases. Furthermore, 15.9% reported being absent from work due to health issues in the previous year. The majority, accounting for 77.1%, described their general health status as good.

**Table 1:** Descriptive characteristics of the participants

	Number (n)	Percentage (%)
Gender (n = 201)		
Male	190	94.5
Female	11	5.5
Age groups (n = 201)		
20-29 years	20	10.0
30-39 years	67	33.3
40-49 years	82	40.8
50 years and older	32	15.9
Graduation status (n = 201)		
Primary school	7	3.5
High school	97	48.3
College	66	32.8
University	21	10.4
Master's/PhD	10	5.0
Total working time (n = 201)		
Less than five years	60	29.9
Five to nine years	44	21.9
10-19 years	50	24.9
20 years and above	47	23.4
Weekly working time (n = 201)		
40 hours	62	30.8
More than 40 hours	139	69.2
Department (n = 201)		
Administrative and social	57	28.4
Technical	144	71.6
Smoking (n = 201)		
Yes	80	39.8
No	121	60.2
Chronic disease (n = 201)		
Yes	40	19.9
No	161	80.1
Absenteeism due to health problems (n = 201)		
Yes	32	15.9
No	169	84.1
Perceived health status (n = 201)		
Very good	31	15.4
Good	124	61.7
Fair	43	21.4
Bad	3	1.5

Table 2 presents the distribution of participant evaluations regarding the work environment. Our findings indicate that 25.4% of the participants reported lacking information about work environment measurements, while 32.3% were unaware of the risk assessment process. In addition, 15.9% stated that periodic maintenance of work equipment was not conducted. Difficulty in obtaining personal protective equipment was mentioned by 11.4% of the participants. A significant 67.2% believed that sufficient measures were not taken against physical risks. Furthermore, 15.4% reported a lack of a designated rest area at work. Negative health impacts from work were noted by 31.8% of the participants. Lastly, 51.2% of the participants identified noise, and 32.8% identified ventilation as physical factors in the work environment that fell outside the acceptable range.

**Table 2:** Participants' evaluations of the working environment

	Number (n)	Percentage (%)
Knowledge about working environment measurements (n = 201)		
Yes	150	74.6
No	51	25.4
Knowledge about risk assessments (n = 201)		
Yes	136	68.2
No	65	32.3
Periodic maintenance of work equipment (n = 201)		
Yes	169	84.6
No	32	15.9
Difficulty in obtaining personal protective equipment (n = 201)		
Yes	23	11.4
No	178	88.6
Adequate precautions are taken against physical risks (n = 201)		
Yes	66	32.8
No	135	67.2
Rest area at work (n = 201)		
Yes	170	84.6
No	31	15.4
Physical factors outside the acceptable range in the work environment (n = 201)		
Noise	103	51.2
Ventilation	66	32.8
Heat	40	19.9
Lighting	35	17.4
Radiation	28	13.9
Vibration	27	13.4
Pressure	5	2.5
Negative health impacts from work (n=201)		
Yes	64	31.8
No	137	68.2

Table 3 displays the levels of work-life conflict and burnout among the participants. The participants reported varying degrees of experiencing work-life conflict: 13.9% reported experiencing it always or often, 17.9% sometimes, 42.8% rarely, and 25.4% almost never or never. Similarly, burnout was reported at different levels: 14.5% always or often, 32.3% sometimes, 32.8% rarely, and 20.4% almost never or never.

**Table 3:** Work-life conflict and burnout levels of the participants

	Number (n)	Percentage (%)
Work-life conflict (n = 201)		
Always/often	28	13.9
Sometimes	36	17.9
Rarely	86	42.8
Almost never/never	51	25.4
Burnout (n = 201)		
Always/often	29	14.5
Sometimes	65	32.3
Rarely	66	32.8
Almost never/never	41	20.4

Table 4 presents the prevalence of work-life conflict and burnout among participants categorized by various demographic characteristics. High work-life conflict was prevalent in 41.8% of individuals aged 30-39, while only 15.6% were observed among those aged 50 and older. Moreover, it was reported that 28.8% of the participants with a high school education or below, contrasting with 35.1% among college graduates or higher. Among employees with less than 10 years of experience, the prevalence stood at 39.4%, whereas it reduced to 23.7% for those with 10 years or more in service. When considering weekly working hours, 33.9% of those working 40 hours per week experienced high work-life conflict compared to 30.9% among those working more than 40 hours. Furthermore, individuals with a negative perceived health status reported a higher prevalence of work-life conflict (50.0%) compared to those with positive perceived health (26.5%). Regarding occupational departments, 24.6% of administrative staff experienced high work-life conflict, whereas it was higher at 34.7% among technical department employees. Significant differences (p < 0.05) in work-life conflict were found across age groups, total working time, and perceived health status. Specifically, individuals aged 20-29 showed the highest prevalence (55.0%) compared to 34.4% among those aged 50 and over. Similarly, participants with higher educational attainment (51.5% among college graduates or more) reported a higher prevalence compared to those with a high school diploma or less (42.3%). Furthermore, individuals with less than 10 years of work experience exhibited a higher prevalence (56.7%) than their counterparts with 10 years or more (36.1%). Similarly, those reporting negative perceived health status (65.2%) had a significantly higher prevalence compared to those with positive perceived health (41.3%). Moving on to burnout levels, 50.9% of administrative department employees and 45.1% of technical department employees experienced high burnout levels. Significant differences (p < 0.05) in burnout were noted concerning total working time, weekly working hours, and perceived health status.

**Table 4:** Work-life conflict and burnout situations according to some characteristics of the participants

	Work-life conflict				Burnout			
	High		Low		High		Low	
	(%)	(n)	(%)	(n)	(%)	(n)	(%)	(n)
Age groups (n = 201)								
20-29 years (n = 20)	10.0	2	90.0	18	55.0	11	45.0	9
30-39 years (n = 67)	41.8	28	58.2	39	49.3	33	50.7	34
40-49 years (n = 82)	35.4	29	64.6	53	47.6	39	52.4	43
50 years and over (n = 32)	15.6	5	84.4	27	34.4	11	65.6	21
	p = 0.008				p = 0.439			
Graduation status (n = 201)								
High school and lower (n = 104)	28.8	30	71.2	74	42.3	44	57.7	60
College and higher(n = 97)	35.1	34	64.9	63	51.5	50	48.5	47
	p = 0.345				p = 0.190			
Total working time (n = 201)								
Less than 10 years (n = 104)	39.4	41	60.6	63	56.7	59	43.3	45
10 years and above (n = 97)	23.7	23	76.3	74	36.1	35	63.9	62
	p = 0.017				p = 0.003			
Weekly working time (n = 201)								
40 hours (n = 62)	33.9	21	66.1	41	58.1	36	41.9	26
More than 40 hours (n = 139)	30.9	43	69.1	96	41.7	58	58.3	81
	p = 0.680				p = 0.032			
Perceived health status (n = 201)								
Positive (n = 155)	26.5	41	73.5	114	41.3	64	58.7	91
Negative (n = 46)	50.0	23	50.0	23	65.2	30	34.8	16
	p = 0.003				p = 0.004			
Department (n = 201)								
Administrative (n = 57)	24.6	14	75.4	43	50.9	29	49.1	28
Technical (n = 144)	34.7	50	65.3	94	45.1	65	54.9	79
	p = 0.163				p = 0.462			

## Discussion

In this cross-sectional study involving 201 employees across three distinct power plants in Turkey, our findings indicate that one in seven employees frequently encounter burnout or struggle with work-life conflict. These results underscore the importance of addressing these issues, as they have the potential to detrimentally impact productivity, motivation, and overall quality of life for employees.

Our research reveals a statistically significant distinction in burnout levels based on the duration of employment, particularly with less than 10 years of service. A study conducted at a power plant in Turkey observed a decrease in burnout as the length of employment increased [14]. Similarly, a study involving employees from an electricity distribution company in Iraq found no notable variance in burnout relative to years of service [15]. Conversely, a study focusing on power plant workers in Iran discovered a lower prevalence of burnout syndrome among those with over 10 years of experience [16]. The absence of a significant distinction in the Iraqi study might be attributed to its limited sample size. Comparing the Iranian research with our own findings, it is evident that the correlation between employment duration and physical burnout aligns in the same direction. Longer tenure in the workforce may lead to a reduced likelihood of burnout, possibly because individuals become adept at managing work demands and crises over time.

In our study, approximately three-fifths of employees with a 40-hour work week reported feeling burned out, while the incidence of physical burnout was two-fifths among employees working more than 40 hours. A study conducted in Taiwan similarly found that employees working over 40 hours per week experienced higher levels of burnout [17]. Another study in the same country corroborated this, demonstrating that those working more than 40 hours experienced increased burnout [18]. Research conducted in Jordan also supports this trend, showing that burnout escalated with weekly working hours [19]. However, our findings contradict this literature, suggesting that burnout does not necessarily increase as weekly working hours rise. It is worth noting that in our study, employees working 40 hours per week were primarily in administrative roles, and perceptions of burnout varied among them.

In this study, approximately 40% of employees who reported positive perceived health experienced burnout due to work, while over 60% of those with poor perceived health status felt burned out. Another study conducted in Turkey found that burnout was less prevalent among individuals with good perceived health status [20]. Similarly, research in Jordan identified a negative correlation between perceived health and burnout [21]. In a study conducted in the United States, a reverse relationship was observed between burnout and perceived health [22]. As perceived health improves, individuals may experience a reduction in burnout, leading to positive effects on their work.

The prevalence of high work-life conflict varies significantly across different demographic groups. For instance, in the 30-39 age group, two-fifths of individuals experience high work-life conflict, while among those aged 50 and older, the prevalence drops to one-sixth. Furthermore, one-fourth of individuals reporting positive perceived health face work-life conflict, whereas half of those reporting negative perceived health encounter this challenge. A nationally representative study conducted in Switzerland discovered that one in eight employees grapples with a high prevalence of work-life conflict. Moreover, this study revealed that employees experiencing work-life conflict are at an elevated risk of reporting poor self-perceived health [23]. Similarly, a study conducted among employees in Turkey revealed that individuals in the 32-38 age group experience more work-family conflict compared to those in the 46-59 age group [24]. These findings underscore the notion that challenges in achieving work-life balance may vary depending on factors such as age, health status, and geographical region.

In our study, roughly three-quarters of the participants reported good to excellent health status, while one-quarter reported fair to poor health status. Comparable findings are noted in research conducted in Turkey, where seven out of 10 employees perceived their health status as good [25]. Another study involving blue-collar workers in Turkey found that three out of four employees rated their health status as good [26]. Similarly, in a study in South Korea, approximately four-fifths of the employees reported good to very good health status [27]. Organisation for Economic Co-operation and Development (OECD) countries also reflect a trend where good perceived health in the general population occurs two-thirds of the time [28]. Employees at hydroelectric power plants have similarly reported a positive perception of their health status, consistent with findings from similar studies in various countries. The healthy worker effect, coupled with the relatively young average age of the employees, could potentially explain these observations.

Based on our findings, approximately three-quarters of the respondents were familiar with work environment measurements, and seven out of 10 were aware of risk assessments. However, only three out of 10 employees in the study believe that adequate measures are taken against physical risk factors in the work environment. Interestingly, despite the majority being aware of work environment measurements and risk assessments, there seems to be a lack of confidence in the adequacy of measures taken against physical risk factors. This may suggest uncertainty regarding the outcomes of work environment measurements and insufficient employee involvement in risk assessments.

In our study, approximately half of the participants reported noise as a concern, while one-third expressed dissatisfaction with ventilation, and one-fifth found the temperature outside the acceptable range in their work environment. A study on noise exposure at a power station in Tanzania found that nearly half of the workers reported hearing loss [29]. Similarly, in a study of electrical services workers in North America, almost two-thirds of participants reported experiencing heat stress, with employees in the electrical and mechanical departments encountering particularly high temperatures [30]. In Nigeria, a quarter of workers noted inadequate ventilation conditions [31]. These findings underscore the significance of noise, ventilation, and thermal comfort as crucial physical risk factors for workers in the hydroelectric industry.

Our study possesses several limitations. There is a male gender bias in the cohort. While the questionnaire encompasses the sub-dimensions of a valid and reliable scale, it is crucial to acknowledge that some data solely rely on employees' self-reporting and lack direct observations or measurements. Notably, due to the typical gender distribution in hydroelectric power plants, comparing genders was unfeasible as the number of female employees was insufficient. Throughout the data collection phase, certain employees remained inaccessible due to factors such as shift work, annual leave, illness, and assignments. Furthermore, a significant number of employees declined participation in the study, citing heavy workloads. However, despite these constraints, our study, comprising 201 participants, stands as one of the most extensive investigations conducted within hydroelectric power plants.

## Conclusions

Hydroelectric power plant workers face significant occupational health and safety risks, with many expressing concerns about inadequate measures to mitigate these risks. Providing regular occupational health and safety training can enhance employees' understanding of these challenges. Moreover, active involvement in risk assessments and transparent sharing of work environment measurements are crucial for addressing employee concerns.

Burnout and work-life conflict are prevalent among hydroelectric power plant employees. To alleviate these issues, several measures can be implemented. First and foremost, scheduling should prioritize regular working hours and discourage overtime, ensuring employees have sufficient rest periods. Fair workload distribution and fostering a supportive work environment are also imperative. Employers should prioritize employees' emotional well-being and provide counseling services to equip them with stress management skills. To boost motivation, it is vital to offer performance rewards, opportunities for career development, and conducive working conditions to enhance job satisfaction. Implementing these measures can potentially reduce burnout and promote a healthier work-life balance for hydroelectric power plant employees.
